# Updating Exercise Testing Strategies and Exercise Prescription Protocols

**DOI:** 10.3390/healthcare12090901

**Published:** 2024-04-26

**Authors:** Rafael Oliveira, João Paulo Brito

**Affiliations:** 1 Sports Science School of Rio Maior, Polytechnic Institute of Santarém, 2040-413 Rio Maior, Portugal; jbrito@esdrm.ipsantarem.pt; 2 Research Centre in Sport Sciences, Health Sciences and Human Development, 5001-801 Vila Real, Portugal

Exercise testing and prescription is still a hot topic. It is evidenced by the constant updating of the guidelines for exercise testing and prescription provided by the American College of Sports Medicine (ACSM). While the guidelines describe the most relevant test for general population with or without specific conditions, it also describes Frequency, Intensity, Time, and Type (FITT) of exercise prescription [1,2].

Another example of this hot topic is the “physical activity on prescription” model adopted by Sweden in which all licensed healthcare professionals may prescribe it. In 2019, a systematic review about the main elements of this model was published. Specifically, the study included studies that compared adults who received this models versus those who did not and the main findings showed that there was a tendency for higher levels of physical activity in those who followed the Swedish model [3]. Additionally, this model was considered the best by the European Commission and it was then used by 10 European countries in a project to implement this approach [4].

Even so, there are several questions that are still not well addressed such as other specific tests in the different contexts, the specific exercises or even questions about training periodization.

Therefore, this Special Issue updated information on exercise testing and prescription strategies to improve quality of life. The present Special Issue contributed to the field with 19 articles:

1. Cabo, C.A.; Fernandes, O.; Mendoza-Muñoz, M.; Barrios-Fernandez, S.; Muñoz-Bermejo, L.; Gómez-Galán, R.; Parraca, J.A. An Active Retirement Programme, a Randomized Controlled Trial of a Sensorimotor Training Programme for Older Adults: A Study Protocol. *Healthcare* **2023**, *11*, 86. https://doi.org/10.3390/healthcare11010086
2. Ferrando-Terradez, I.; Dueñas, L.; Parčina, I.; Ćopić, N.; Petronijević, S.; Beltrami, G.; Pezzoni, F.; San Martín-Valenzuela, C.; Gijssel, M.; Moliterni, S.; et al. Women’s Involvement in Steady Exercise (WISE): Study Protocol for a Randomized Controlled Trial. *Healthcare* **2023**, *11*, 1279. https://doi.org/10.3390/healthcare11091279
3. Sousa, M.; Oliveira, R.; Brito, J.P.; Martins, A.D.; Moutão, J.; Alves, S. Effects of Combined Training Programs in Individuals with Fibromyalgia: A Systematic Review. *Healthcare* **2023**, *11*, 1708. https://doi.org/10.3390/healthcare11121708
4. Denche-Zamorano, Á.; Mendoza-Muñoz, D.M.; Barrios-Fernandez, S.; Perez-Corraliza, C.; Franco-García, J.M.; Carlos-Vivas, J.; Pastor-Cisneros, R.; Mendoza-Muñoz, M. Physical Activity Reduces the Risk of Developing Diabetes and Diabetes Medication Use. *Healthcare* **2022**, *10*, 2479. https://doi.org/10.3390/healthcare10122479
5. Muñoz-Paredes, I.; Herrero, A.J.; Román-Nieto, N.; Peña-Gomez, A.M.; Seco-Calvo, J. Influence of Transcranial Direct Current Stimulation and Exercise on Fatigue and Quality of Life in Multiple Sclerosis. *Healthcare* **2023**, *11*, 84. https://doi.org/10.3390/healthcare11010084
6. Hsu, W.-H.; Hsu, W.-B.; Lin, Z.-R.; Chang, S.-H.; Fan, C.-H.; Kuo, L.-T.; Hsu, W.-W.R. Effects of 24 Weeks of a Supervised Walk Training on Knee Muscle Strength and Quality of Life in Older Female Total Knee Arthroplasty: A Retrospective Cohort Study. *Healthcare* **2023**, *11*, 356. https://doi.org/10.3390/healthcare11030356
7. De Araújo Moury Fernandes, G.C.; Barbosa Junior, J.G.G.; Seffrin, A.; Vivan, L.; de Lira, C.A.B.; Vancini, R.L.; Weiss, K.; Knechtle, B.; Andrade, M.S. Amateur Female Athletes Perform the Running Split of a Triathlon Race at Higher Relative Intensity than the Male Athletes: A Cross-Sectional Study. *Healthcare* **2023**, *11*, 418. https://doi.org/10.3390/healthcare11030418
8. So, B.C.L.; Kwok, M.M.Y.; Lee, N.W.L.; Lam, A.W.C.; Lau, A.L.M.; Lam, A.S.L.; Chan, P.W.Y.; Ng, S.S.M. Lower Limb Muscles’ Activation during Ascending and Descending a Single Step-Up Movement: Comparison between In water and On land Exercise at Different Step Cadences in Young Injury-Free Adults. *Healthcare* **2023**, *11*, 441. https://doi.org/10.3390/healthcare11030441
9. Ma, P.-S.; So, W.-Y.; Choi, H. Using the Health Belief Model to Assess the Physical Exercise Behaviors of International Students in South Korea during the Pandemic. *Healthcare* **2023**, *11*, 469. https://doi.org/10.3390/healthcare11040469
10. Pérez-Quero, F.J.; Granero-Gallegos, A.; Baena-Extremera, A.; Baños, R. Goal Orientations of Secondary School Students and Their Intention to Practise Physical Activity in Their Leisure Time: Mediation of Physical Education Importance and Satisfaction. *Healthcare* **2023**, *11*, 568. https://doi.org/10.3390/healthcare11040568
11. Reljic, D.; Frenk, F.; Herrmann, H.J.; Neurath, M.F.; Zopf, Y. Maximum Heart Rate- and Lactate Threshold-Based Low-Volume High-Intensity Interval Training Prescriptions Provide Similar Health Benefits in Metabolic Syndrome Patients. *Healthcare* **2023**, *11*, 711. https://doi.org/10.3390/healthcare11050711
12. Stephan, H.; Wehmeier, U.F.; Förster, T.; Tomschi, F.; Hilberg, T. Additional Active Movements Are Not Required for Strength Gains in the Untrained during Short-Term Whole-Body Electromyostimulation Training. *Healthcare* **2023**, *11*, 741. https://doi.org/10.3390/healthcare11050741
13. Amado, B.L.; De Lira, C.A.B.; Vancini, R.L.; Forte, P.; Costa, T.; Weiss, K.; Knechtle, B.; Andrade, M.S. Comparison of Knee Muscular Strength Balance among Pre- and Post-Puberty Adolescent Swimmers: A Cross-Sectional Pilot Study. *Healthcare* **2023**, *11*, 744. https://doi.org/10.3390/healthcare11050744
14. Mahfouz, M.S.; Alqassim, A.Y.; Sobaikhi, N.H.; Jathmi, A.S.; Alsadi, F.O.; Alqahtani, A.M.; Shajri, M.M.; Sabi, I.D.; Wafi, A.M.; Sinclair, J. Physical Activity, Mental Health, and Quality of Life among School Students in the Jazan Region of Saudi Arabia: A Cross-Sectional Survey When Returning to School after the COVID-19 Pandemic. *Healthcare* **2023**, *11*, 974. https://doi.org/10.3390/healthcare11070974
15. Lavín-Pérez, A.M.; León-Llamas, J.L.; Salas Costilla, F.J.; Collado-Mateo, D.; López de las Heras, R.; Gasque Celma, P.; Villafaina, S. Validity of On-Line Supervised Fitness Tests in People with Low Back Pain. *Healthcare* **2023**, *11*, 1019. https://doi.org/10.3390/healthcare11071019
16. Tan, J.; Krasilshchikov, O.; Kuan, G.; Hashim, H.A.; Aldhahi, M.I.; Al-Mhanna, S.B.; Badicu, G. The Effects of Combining Aerobic and Heavy Resistance Training on Body Composition, Muscle Hypertrophy, and Exercise Satisfaction in Physically Active Adults. *Healthcare* **2023**, *11*, 2443. https://doi.org/10.3390/healthcare11172443
17. Brites-Lagos, C.; Ramos, L.; Szumilewicz, A.; Santos-Rocha, R. Feasibility of a Supervised Postpartum Exercise Program and Effects on Maternal Health and Fitness Parameters—Pilot Study. *Healthcare* **2023**, *11*, 2801. https://doi.org/10.3390/healthcare11202801
18. Ma, X.; Yan, J.; Liu, W. An Early Indicator in Evaluating Cardiac Dysfunction Related to Premature Ventricular Complexes: Cardiorespiratory Capacity. *Healthcare* **2023**, *11*, 2940. https://doi.org/10.3390/healthcare11222940
19. Rodríguez-García, L.; Ceylan, H.I.; Silva, R.M.; Silva, A.F.; Guadalupe-Grau, A.; Liñán-González, A. Effects of 10-Week Online Moderate- to High-Intensity Interval Training on Body Composition, and Aerobic and Anaerobic Performance during the COVID-19 Lockdown. *Healthcare* **2024**, *12*, 37. https://doi.org/10.3390/healthcare12010037

In resume, the articles addressed at least one dimension of physical fitness (cardiorespiratory endurance, body composition, muscular strength, muscular endurance, and flexibility) as well as quality of life, mainly measured by questionnaires (see Table 1). Moreover, it is relevant to highlight that this special issue also included studies that addressed specific conditions such as fibromyalgia, multiple sclerosis, knee arthroplasty, obesity with metabolic syndrome, low back pain, postpartum women, and people with premature ventricular beats.

The current special issue provided and constitutes relevant information for fitness professional and exercise physiologists. At the same time, it showed meaningful findings about the online exercise testing procedures (see contributors 15 and 17). In addition, it was observed that there are still several research that uses only one type of exercise, despite the general guidelines of the ACSM recommend more that one type [1]. Furthermore, there is a recommendation for future research include behavior change theories in exercise intervention as suggested by the protocol of the contributor 2. Finally, the present special issue also reinforces more research on specific populations, with different ways to control intensity, more specialized tests, while including training periodization practices as well as behavior changes.

## Figures and Tables

**Table 1:** Analysis of the published contributions in the Special Issue.

Contributor Number	Target Population	Study Type	Exercise Testing	Exercise Type
1	Older adults	Protocol	Body composition, physical fitness, and questionnaires: health related quality of life; physical activity level	Cardiorespiratory and strength
2	Women adults	Protocol	Daily steps, program adherence, anthropometry, body composition, plank test, 6-min walking test; questionnaires: International Physical Activity; healthy lifestyle and personal control; Pittsburgh Sleep Quality Index; visual analogue scale; physical activity enjoyment scale	Cardiorespiratory (HIIT) training and behavior change theories and techniques.
3	People with fibromyalgia	Systematic Review	Several instruments/tests were used to assess pain, sleep quality, health status and strength gains in the upper and lower limbs	Combined training, HIIT, Tai Chi, aerobic exercise, body balance and strength
4	Participants from 15 to 69 years	Cross-sectional	Diabetes prevalence and Diabetes medication use in diabetics, Physical Activity Level, Body Mass Index, Spanish National Health Survey	NA
5	People with multiple sclerosis	Cross-over	Questionnaires: Multiple Sclerosis International Quality of Life; International Physical Activity; Kurtzke Expanded Disability Status Scale; Spanish version of the Modified Fatigue Impact Scale	Strength and aerobic
6	Older women with knee arthroplasty	Cohort	Isokinetic strength, 6-min walk test, the 8-foot up-and-go test, and the 30-s chair stand test, Knee Injury and Osteoarthritis Outcome Score (KOOS) Questionnaire	Cardiorespiratory exercise
7	Amateur male and female adult athletes	Cross-sectional	Cardiopulmonary exercise test	NA
8	Young adults	Cross-sectional	Muscle activation in water and land step exercise	NA
9	Adults	Cross-sectional	Health belief model (questionnaire)	NA
10	Young (from 12 to 19 years)	Cross-sectional	Questionnaires: Perception of Success; Importance of Physical Education; Satisfaction with Physical Education Intention to partake in leisure time physical activity.	NA
11	Obese people with metabolic syndrome	Cohort	Hydration, blood, anthropometric, cardiopulmonary exercise test, quality of life (questionnaire), daily nutrition.	Cardiorespiratory (HIIT)
12	Adults	Cohort		Strength with electromyostimulation
13	Adolescent swimmers	Cross-sectional	Sexual maturity, body composition, isokinetic strength test	NA
14	Young (from 12 to 18 years)	Cross-sectional	Questionnaires: Fels PAQ; Depression Anxiety Stress Scales; Pediatric Quality of Life Inventory	NA
15	Adult and older (45–72 years) with low back pain	Cross-sectional	Online evaluations: 30-s chair stand-up test; arm curl test; 2-min step test in place; chair-stand and reach test; back scratch; 8-foot up-and-go test; Sharpened Romberg; one-legged stance test	NA
16	Adults	Cohort	body composition, muscle hypertrophy, and exercise satisfaction	Combined aerobic and strength
17	Postpartum women	Cohort pilot study	Blood pressure; anthropometry, body composition; chair stand test; cardiopulmonary exercise test, push-up test; V-sit and reach. Questionnaires: Physical Activity Readiness Questionnaire for Everyone; International Physical Activity; World Health Organization Quality of Life Questionnaire; Pelvic Girdle; Roland-Morris Disability; Fatigue Assessment Scale; Edinburgh Postpartum Depression Scale	Combined training: cardiorespiratory, postural, functional/resistance training, neuromotor training, stretching, breathing, and relaxation exercises
18	People with premature ventricular beats	Cross-sectional	Cardiopulmonary exercise test	NA
19	Women adults	Cohort	Cardiopulmonary exercise test	Cardiorespiratory (HIIT)

HIIT, high-intensity interval training; NA, non-applicable.
